# Diabetes Distress During COVID-19: Three Brief ‘Snapshot’ Surveys of Adults With Diabetes Calling the Australian National Diabetes Services Scheme Helpline

**DOI:** 10.3389/fcdhc.2021.769528

**Published:** 2021-12-24

**Authors:** Edith E. Holloway, Christel Hendrieckx, George Company, Timothy C. Skinner, Jane Speight

**Affiliations:** ^1^ School of Psychology, Deakin University, Geelong, VIC, Australia; ^2^ The Australian Centre for Behavioural Research in Diabetes, Diabetes Victoria, Melbourne, VIC, Australia; ^3^ Diabetes Victoria, Melbourne, VIC, Australia; ^4^ La Trobe Rural Health School, La Trobe University, Flora Hill, VIC, Australia; ^5^ Department of Psychology, University of Copenhagen, Copenhagen, Denmark

**Keywords:** diabetes, diabetes distress, emotional wellbeing, COVID-19, mental health, survey

## Abstract

The aim of this study was to take ‘snapshots’ of how people with diabetes are feeling emotionally during the coronavirus disease 2019 (COVID-19) pandemic. Three ‘snapshot’ surveys were conducted during May 2020, August 2020 and April 2021, each over a two-week period. Adults (≥18 years) with diabetes calling the Australian Government’s National Diabetes Services Scheme Helpline (NDSS) were invited to participate. Those who accepted were asked three questions sourced/adapted from the Problem Areas in Diabetes scale. Responses were recorded on a 5-point scale (0=’not a problem’, 4=’serious problem’). Of interest were scores ≥2, indicating this was at least a ‘moderate problem’. The survey was administered by NDSS Helpline staff *via* telephone. Basic demographic and clinical data were collected. In total, 1,278 surveys were completed over the three ‘snapshots’ (1^st^ N=449; 2^nd^ N=414; 3^rd^ N=415). Participants were aged (median[IQR]) 62[47,72] years, 56% were women, and 57% had type 2 diabetes. At the 3rd ‘snapshot’, 21% had received a COVID-19 vaccine. Our findings show that feeling at least moderately ‘burned out’ by the constant effort needed to manage diabetes is salient, and consistently experienced by adults with diabetes calling the NDSS Helpline at three timepoints during the coronavirus pandemic. Those who participated in the 3^rd^ ‘snapshot’ survey were less likely to report that feeling ‘alone with their diabetes’ or ‘worrying about their diabetes because of the COVID-19 pandemic’ were moderate or serious problems for them. Except for younger adults, findings indicate that the easing of restrictions may mitigate some of the effects of the pandemic on diabetes-specific emotional problems, including feeling ‘burned out’, ‘alone’ with diabetes, and/or worried about diabetes due to COVID-19. Prospective data are needed to improve our understanding of the emotional impact of COVID-19 on people with diabetes and to inform when and how to target support for those who need it most.

## Introduction

The novel coronavirus disease (COVID-19) is associated with serious physical consequences (1). Mounting evidence highlights that the pandemic (and associated restrictions to personal freedoms) is having a detrimental impact on mental health in the general population (2). For people with diabetes, COVID-19 infection is associated with increased risk of serious complications and/or death (3, 4), while the pandemic and associated restrictions contribute to increased distress, stigmatisation and social isolation (5). Changes in the number of active cases in the community, levels of restrictions, and vaccination rates, may impact how people are coping with, and adapting to, the challenges of this novel virus at any given point in time.

Our aim was to obtain brief snapshots of how adults are feeling about their diabetes at three timepoints during the COVID-19 pandemic. Prior to the first ‘snapshot’ survey period (26 May to 9 June 2020), pandemic restrictions had been in place but were about to be eased nationally. During the second ‘snapshot’ (20 August to 3 September 2020), Melbourne had widespread community transmission, and a second lockdown, while all other states reported zero or few cases and minimal restrictions. During the third ‘snapshot’ (27 April to 13 May 2021), most states had no community transmission and minimal restrictions. Furthermore, the Australian Government’s COVID-19 vaccination program had commenced, though only 2.9 million doses (11 per 100 people) had been administered at that time (6).

## Materials and Methods 

We conducted three cross-sectional ‘snapshot’ surveys of diabetes-specific distress, associated with feeling ‘burned out’, alone and worried because of the COVID-19 pandemic, among inbound callers to the National Diabetes Services Scheme (NDSS) Helpline. The NDSS Helpline service provides practical support for people with diabetes, including advice on diabetes self-management and information about NDSS services and products. Each ‘snapshot’ survey was conducted over two-week periods commencing May and August 2020 and April 2021. Adults (≥18 years) with diabetes calling the NDSS Helpline (for any reason) were invited to take part in the brief telephone surveys.

Three brief questions were sourced or adapted from the Problem Areas in Diabetes (PAID) scale (7). Respondents were asked, “Which of the following are currently a problem for you?”: a) Feeling ‘burned out’ by the constant effort needed to manage diabetes? b) Feeling alone with your diabetes? c) Worrying about your diabetes because of the COVID-19 pandemic? Participants rated each item (from 0=not a problem, to 4=serious problem). The three items demonstrated acceptable internal consistency (Cronbach's α=0.733). These items were selected given the salience of these issues for people with type 1 diabetes (T1D) and type 2 diabetes (T2D) (7, 8), particularly during the COVID-19 pandemic (9, 10); and also because NDSS factsheets offering support for these issues were readily available for those experiencing problems in these areas (11). Thus, if the caller’s responses indicated that any item was a moderate or serious problem (score ≥2), NDSS Helpline call operators recommended relevant NDSS emotional health factsheets (8): ‘Diabetes distress’ (item 1), ‘Peer support’ (item 2), and ‘Managing worry about COVID-19 and diabetes” (item 3). The factsheets were recommended in addition to usual referrals and support suggested by Helpline staff, including the offer of referral to an ‘on-call health professional’.

In addition to the three survey items, for each participant, the following data were recorded: NDSS registration number, age, gender, postcode, diabetes type and, for those with T2D: treatment type (typically, insulin versus non-insulin). Participants who completed the 2^nd^ and 3^rd^ ‘snapshots’ were asked if they had taken part in the 1^st^ or 2^nd^ surveys. Unique to the 3^rd^ ‘snapshot’ survey, participants were also asked if they had received a COVID-19 vaccine.

### Statistical Analysis

We examined the proportion of participants, by diabetes type, who reported scores of ≥2, indicating the item was at least a moderate problem, at each time point. We examined scores ≥2 given that moderate levels of diabetes distress are regarded to be clinically significant in the scoring of diabetes distress measures and can have a significant impact on how a person manages their diabetes (3, 4, 7, 8). Differences between timepoints were analysed using Pearson Chi-square tests or Fisher’s exact tests. Between the three timepoints, item scores (0-4) were also compared using Kruskal-Wallis tests. *Post-hoc* pairwise tests with Bonferroni correction were performed on categories with significant (*P<*0.05) Kruskal-Wallis results.

All tests were 2-sided, with p<0.05 considered statistically significant. Analyses were performed using SPSS v26.

## Results

During the 2-week survey periods, the NDSS Helpline received N=5,932 inbound calls from people with diabetes: n=2,168 (1^st^), n=2,119 (2^nd^) and n=1,645 (3^rd^). Of these callers, 479 (22%), 454 (21%) and 451 (27%) were invited to participate, respectively in the 1^st^, 2^nd^ and 3^rd^ ‘snapshot’ surveys. In total, N=1,278 surveys were completed over the three ‘snapshots’ (1^st^ N=449; 2^nd^ N=414; 3^rd^ N=415) by 1,248 eligible adults (n=30 completed a survey at 2 timepoints), representing a 94%, 92% and 93% acceptance rate among those invited. Demographic and clinical characteristics were similar across timepoints (**Table 1**). At the 3^rd^ ‘snapshot’, 21% (n=88) had received a COVID-19 vaccine dose. There were no differences (by gender, state or diabetes type) between those who had and had not received the vaccine. Of those who received a vaccine dose, 50% were >70 years old and 39% were 50-69 years old.

**Table 1:** Participants’ demographic and clinical characteristics in the 1^st^ (May 2020), 2^nd^ (August 2020) and 3^rd^ (April 2021) ‘snapshot’ surveys.

Participant characteristic	1^st^ snapshot (N = 449)	2^nd^ snapshot (N = 414)	3^rd^ snapshot (N = 415)
			
**Age, years^#^** 18-34 years 35-49 years 50-69 years ≥70 years	60+16.99, 19-93 63 [49, 73] 48 (11) 68 (15) 167 (37) 166 (37)	60+16.68, 19-91 62 [47, 72] 46 (11) 70 (17) 156 (38) 138 (33)	58+16.92,18-90 61 [44, 72] 53 (13) 80 (19) 159 (38) 123 (30)
**Gender**: Women	249 (56)	234 (56)	237 (57)
**State^~^**			
Australian Capital Territory (ACT) New South Wales (NSW) Northern Territory (NT) Queensland (QLD) South Australia (SA) Tasmania (TAS) Victoria (VIC) Western Australian (WA)	1 (<1) 129 (29) 1 (<1) 111 (25) 22 (5) 9 (2) 146 (33) 25 (6)	7 (2) 116 (28) 0 (0) 74 (18) 22 (5) 5 (1) 168 (41) 19 (5)	7 (2) 136 (33) 2 (<1) 89 (22) 30 (7) 7 (2) 118 (29) 23 (6)
**Diabetes type**			
Type 1 diabetes Type 2 diabetes Gestational diabetes Other or unknown	149 (33) 279 (62) 14 (3) 7 (2)	150 (36) 252 (61) 11 (3) 1 (<1)	215 (52) 192 (46) 7 (2) 1 (<1)
**Treatment type (Type 2 diabetes only)^^^**			
Insulin Non-insulin	77 (28) 197 (71)	98 (39) 153 (61)	45 (24) 146 (76)

Data are n (%) or mean±SD, range or median [IQR].

^#^Age: data missing for 4 participants at 2nd survey.

~State: data missing for 5 participants (1^st^ survey), 3 participants (2^nd^ survey), 3 participants (3^rd^ survey).

^Treatment type: data missing for 5 participants (1^st^ survey), 1 participant (2^nd^ survey), 1 participant (3^rd^ survey) with type 2 diabetes.

During the 1^st^, 2^nd^ and 3^rd^ ‘snapshots’, 42% (n=530) of respondents reported diabetes-specific distress related to at least one problem (i.e. score ≥2 on at least one survey item). This differed between timepoints: 48% in the 2^nd^ survey, compared to 40% in the 1^st^ and 37% in the 3^rd^ ‘snapshot’ (p=0.004). More participants in the 2nd ‘snapshot’ reported that all three survey items were at least a moderate problem (11%) compared to participants in the 1st (8%) and 3rd (6%) ‘snapshots’ (p=0.029).

### Feeling ‘Burned Out’

During the 1^st^, 2^nd^ and 3^rd^ ‘snapshots’, 30%, 41% and 38% of adults with T1D and 24%, 29% and 23% of adults with T2D, respectively reported feeling at least moderately ‘burned out’ by the constant effort needed to manage their diabetes (**Table 2**). Item scores were not significantly different between timepoints (Kruskal–Wallis test: T1D, p=0.476, T2D, p=0.316).

**Table 2:** Proportion of participants with type 1 diabetes and type 2 diabetes who reported each issue as ‘a moderate-to-serious’ problem during the 1^st^ (May 2020), 2^nd^ (August 2020) and 3^rd^ (April 2021) ‘snapshot’ surveys.

Survey item	Diabetes type	1^st^ ‘snapshot’ (N=428)	2^nd^ ‘snapshot’ (N=402)	3^rd^ ‘snapshot’ (N=407)	*χ2 P*-value	Kruskal-Wallis *P*-value^#^	*Post-hoc* test^~^ (*P*- value)		
							*1^st^ vs. 2^nd^*	1^st^ vs. 3^rd^	2^nd^ vs 3^rd^
1. ‘Burned out’ by diabetes	T1D T2D Total	45 (30.2) 66 (23.7) 111 (25.9)	61 (40.7) 73 (29.0) 134 (33.1)	81 (37.7) 45 (23.4) 126 (31.0)	0.149 0.283 0.059	0.476 0.316 0.402	- - -	- - -	- - -
2. Feeling alone with diabetes	T1D T2D Total	38 (25.5) 49 (17.6) 87 (20.3)	39 (26.0) 47 (18.7) 86 (21.4)	34 (15.8) 16 (8.3) 50 (12.3)	0.026 0.006 0.001	0.044 0.020 0.005	0.941 0.834 0.971	0.033 0.010 0.004	0.040 0.019 0.006
3. Worried about diabetes due to COVID-19	T1D T2D Total	43 (28.9) 62 (22.2) 105 (24.5)	57 (38.0) 69 (27.4) 126 (31.3))	38 (17.8) 21 (10.9) 59 (14.5)	<0.001 <0.001 <0.001	<0.001 0.001 <0.001	0.154 0.344 0.072	0.014 0.003 0.001	p<0.001 p<0.001 p<0.001
Total diabetes distress score^*	T1D T2D Total	2.00 (0.5-5) 2.00 (0-4) 2.00 (0-4)	3.00 (0-6) 2.00 (0-4) 2.00 (0-5)	2.00 (0-4) 1.00 (0-3) 1.00 (0.3)	- - -	0.022 0.002 0.001	0.247 0.405 0.137	0.140 0.006 0.027	0.006 0.001 <0.001

Data are n (%) for proportion of participants who reported item was at least a moderate problem (score≥2 on 0-4 scale).

^median [interquartile range].

*Total distress score is the sum of all three survey items (ranging from 0 to 12).

^#^Kruskal-Wallis test was used to compare differences between the three ‘snapshots’ on the 0-4 scale.

~Pairwise comparisons were conducted adjusted by the Bonferroni correction.

### Feeling Alone With Diabetes

During the 1^st^, 2^nd^ and 3^rd^ ‘snapshots’, 26%, 26% and 16% of participants with T1D, and 17%, 18% and 8% of adults with T2D, respectively, reported feeling ‘alone’ with diabetes was at least a moderate problem for them (Kruskal–Wallis test: T1D, p=0.044, T2D, p=0.020). *Post-hoc* comparisons, adjusted by the Bonferroni correction comparing differences in item scores between timepoints (**Table 2**) found that feeling ‘alone with your diabetes’ was less problematic at the 3^rd^ ‘snapshot’ compared to the 1^st^ (T1D, p=0.033; T2D, p=0.010) and 2^nd^ (T1D, p=0.040; T2D, p=0.019).

### Worried About Diabetes Because of COVID-19

During the 1^st^, 2^nd^ and 3^rd^ ‘snapshots’, 29%, 38% and 18% of participants with T1D, and 22%, 27% and 11% of adults with T2D, respectively, reported feeling at least moderately ‘worried about their diabetes because of COVID-19’ (Kruskal–Wallis test: T1D, p<0.001, T2D, p=0.001). *Post-hoc* comparisons, adjusted by the Bonferroni correction comparing differences in item scores between timepoints found that ‘worrying about diabetes because of COVID-19’ was less problematic at the 3^rd^ ‘snapshot’ compared to the 1^st^ (T1D, p=0.014; T2D, p=0.003) and 2^nd^ (T1D, p<0.001; T2D, p<0.001) (**Table 2**).

### Differences by Demographic Characteristics

Younger participants (18-34 years) were more likely to report feeling at least moderately ‘burned out’ during the 3^rd^ ‘snapshot’ (47%), compared to 17% and 30% during the 1^st^ and 2^nd^ surveys, respectively (p=0.004; **Table 3**).

**Table 3:** Proportion of participants with T1D and T2D during the 1^st^ (May 2020), 2^nd^ (August 2020) and 3^rd^ (April 2021) ‘snapshot’ surveys who reported each item as ‘a moderate-to-serious’ problem (score ≥2), by type of diabetes and demographic characteristics.

Survey item	‘Burned out’ by diabetes				Feeling alone with diabetes				Worried about diabetes due to COVID-19			
‘Snapshot’ period	1^st^ n (%)	2^nd^ n (%)	3^rd^ n (%)	*P*-value	1^st^ n (%)	2^nd^ n (%)	3^rd^ n (%)	*P*-value	1^st^ n (%)	2^nd^ n (%)	3^rd^ n (%)	*P*-value
**Type 1 diabetes**												
**Age**												
18-35 years 35-49 years 50-69 years ≥70 years	5 (17) 12 (41) 20 (38) 6 (19)	11 (31) 21 (64) 21 (40) 8 (29)	23 (49) 23 (45) 28 (40) 7 (15)	**0.013** 0.150 0.955 0.355	3 (10) 9 (31) 18 (34) 8 (26)	7 (20) 10 (30) 16 (31) 6 (21)	13 (28) 11 (22) 4 (6) 6 (13)	0.173 0.553 **<0.001** 0.327	4 (13) 8 (28) 24 (45) 6 (19)	12 (34) 12 (36) 27(52) 6 (21)	11 (23) 12 (24) 9 (13) 6 (13)	0.142 0.441 **<0.001** 0.571
**Gender**												
Men Women	18 (27) 27 (33)	24 (39) 37 (42)	28 (30) 53 (44)	0.282 0.283	16 (24) 22 (27)	14 (23) 25 (28)	11 (12) 23 (19)	0.083 0.243	15 (22) 28 (34)	22 (36) 35 (39)	12 (13) 26 (22)	**0.003** **0.015**
State*												
NSW QLD VIC	16 (35) 14 (38) 9 (20)	19 (39) 14 (54) 19 (36)	20 (32) 22 (45) 23 (37)	0.741 0.453 0.120	12 (26) 11 (30) 11 (24)	9 (18) 10 (39) 14 (26)	9 (14) 9 (18) 12 (19)	0.297 0.154 0.629	16 (35) 8 (22) 12 (26)	35 (37) 26 (35) 24 (45)	11 (18) 9 (19) 15 (24)	**0.043** 0.291 **0.030**
**Type 2 diabetes**												
**Age**												
18-35 years 35-49 years 50-69 years ≥70 years	1 (25) 3 (10) 29 (27) 30 (23)	3(38) 10 (35) 40 (39) 18 (16)	2 (100) 5 (19) 25 (28) 13 (17)	0.194 0.068 0.150 0.397	1 (25 1 (3) 20 (19) 24 (18)	2 (25) 6 (21) 29 (28) 8 (7)	0 (0) 1 (4) 12(14) 3 (4)	0.727 **0.037** **0.041** **0.002**	0 (0) 7 (23) 28 (26) 25 (19)	2(25) 9 (31) 37 (36) 20 (18)	0 (0) 3 (12) 15 (17) 3 (4)	0.417 0.220 **0.013** **0.008**
**Gender**												
Men Women	27 (21) 39 (27)	33 (28) 40 (30)	18 (22) 27 (25)	0.364 0.634	19 (14) 30 (20)	17 (14) 30 (23)	7 (8) 9 (8)	0.379 **0.008**	22 (17) 40 (27)	30 (25) 39 (29)	9 (11) 12 (11)	**0.028** **0.001**
State*												
NSW QLD VIC	17 (22) 13 (19) 25 (26)	16 (25) 11 (24) 35 (32)	16 (23) 12 (32) 10 (19)	0.863 0.351 0.193	13 (17) 8 (12) 21 (22)	11 (18) 6 (13) 22 (20)	5 (17) 6 (16) 3 (6)	0.159 0.841 **0.029**	15 (19) 14 (21) 25 (26)	14 (22) 12 (26) 35 (32)	7 (10) 8 (21) 4 (7)	0.155 0.768 **0.003**
**Treatment type**												
Insulin Non-insulin	22 (29) 43 (22)	35 (36) 37 (24)	16 (36) 29 (20)	0.568 0.665	19 (25) 30 (15)	20 (20) 27 (18)	8 (18) 7 (5)	0.638 **0.002**	21 (27) 39 (20)	32 (33) 37 (24)	8 (18) 13 (9)	0.181 **0.002**

*Respondents from New South Wales (NSW), Queensland (QLD) and Victoria (VIC) comprise 84-87% of the sample across timepoints. Due to small numbers, respondents were excluded from these analyses from the following states and territories: Australian Capital Territory (ACT), Northern Territory (NT), South Australia (SA), Tasmania (TAS), Western Australian (WA).

Items in bold are considered statistically significant.

Overall, men and women with T1D and T2D reported feeling less worried about their diabetes due to COVID-19 during the 3^rd^ ‘snapshot’ compared with earlier surveys (**Table 3**). However, during the 1^st^ ‘snapshot’, women with T2D were more likely to report feeling worried about their diabetes due to COVID-19 compared with men (p=0.043).

During the 3^rd^ survey, there was a trend towards a higher proportion of women with T1D feeling at least moderately ‘burned out ‘compared with men (p=0.047).

Within state comparisons over time showed that feeling at least moderately worried about diabetes due to COVID-19 was more likely at the 2^nd^ than the 3^rd^ ‘snapshot’ among participants residing in New South Wales (2^nd^: 28% vs 3^rd^: 13%; p=0.008) and Victoria (2^nd^: 35% vs 3^rd^: 16%; p=0.001). No significant differences were found between the states on any of the three survey items.

At the 3rd ‘snapshot’, no significant differences were found on any of the survey items by vaccination status among adults with T1D and T2D (**Table 4**).

**Table 4:** Proportion of participants who reported each issue as ‘a moderate-to-serious’ problem during the 3^rd^ ‘snapshot’ survey (April 2021), by diabetes type and vaccination status.

Survey item	Diabetes type	Vaccinated^ (N = 88)	Unvaccinated (N = 327)	*χ2 P*-value	Mann-Whitney* *P*-value
1. ‘Burned out’ by diabetes	T1D T2D Total	18 (40.9) 7 (15.9) 25 (28.4)	63 (36.8) 38 (25.7) 101 (31.7)	0.620 0.179 0.559	0.844 0.063 0.157
2. Feeling alone with diabetes	T1D T2D Total	4 (9.1) 1 (2.3) 5 (5.7)	30 (17.5) 15 (10.1) 45 (14.1)	0.246 0.125 **0.042**	0.320 0.068 0.066
3. Worried about diabetes due to COVID-19	T1D T2D Total	7 (15.9) 2 (4.5) 9 (10.2)	31 (18.2) 19 (12.8) 50 (15.7)	0.827 0.170 0.233	0.846 0.079 0.345

Data are n (%) for the proportion of participants who reported item being at least a moderate problem (score ≥2).

^Vaccinated means at least (and typically) one vaccine dose.

*Mann-Whitney Test was used to compare differences between vaccinated vs. unvaccinated groups on the 0-4 scale.

Items in bold are considered statistically significant.

**Table 5** summarises the rate of acceptance of relevant NDSS factsheets and uptake of a referral to an ‘on-call’ diabetes health professional (where item scores indicated at least a moderate problem). The rates of uptake when participants were offered a relevant NDSS factsheet were lower during the 3^rd^ survey compared to the previous two surveys (p=0.022). Rates of uptake when participants were offered a referral to an ‘on-call’ health professional were comparable across surveys (p=0.064).

**Table 5:** Referral type and uptake by participants who reported ‘moderate-to-severe diabetes distress’ in the 1^st^, 2^nd^ and 3^rd^ ‘snapshot’ surveys.

Type of support offered	1st Snapshot (N = 180)		2nd Snapshot (N = 198)		3rd Snapshot (N = 152)	
	Offered	Accepted	Offered	Accepted	Offered	Accepted
	n (%)	n (%)	n (%)	n (%)	n (%)	n (%)
Health professional referral	136 (76)	40 (29)	148 (75)	47 (32)	141 (93)	31 (22)
NDSS factsheet(s)	117 (65)	65 (56)	132 (67)	65 (49)	120 (79)	37 (31)
Diabetes distress	72/116 (62)	39 (54)	91/132 (69)	48 (53)	101/126 (80)	35 (35)
Peer support	57/88 (65)	36 (63)	61/87 (70)	31 (51)	44/50 (88)	19 (43)
Managing worry zabout diabetes and COVID-19	77/109 (71)	48 (62)	88/128 (69)	45 (51)	48/59 (81)	19 (40)

1st snapshot (May) survey: 180 participants had a score of >2 on at least one survey item; 2nd snapshot (August) survey: 198 participants had a score of >2 on at least one survey item; 3rd snapshot (April) survey: 152 participants had a score of >2 on at least one survey item.

## Discussion

Our findings demonstrate that adults calling the NDSS Helpline at three time-points during the COVID-19 pandemic were at least moderately distressed about their diabetes. On average, 42% (n=530) of respondents reported that at least one of the three issues explored was a moderate-to-severe problem for them. Participants were more likely to report feeling ‘burned out’, alone and worried about their diabetes because of COVID-19 during the 2^nd^ ‘snapshot’ compared to other timepoints. During the 2^nd^ ‘snapshot’, 6 months into the pandemic, a second lockdown was imposed in Melbourne (with some restrictions at a state level in Victoria). The state was also experiencing a peak number of daily new infections compared to the other timepoints.

Our findings suggest that, during the first year of the COVID-19 pandemic, adults with diabetes are more likely to report feeling ‘burned out’ and ‘alone with their diabetes’, compared with pre-COVID-19 levels (8, 12–14). There is a paucity of data reporting on the longitudinal impact of the COVID-19 pandemic on diabetes distress. A longitudinal study of adults with diabetes in Denmark, found that diabetes distress and general loneliness reduced three months into the COVID-19 pandemic. Similarly, compared to 6 and 12 months prior, participants completing the 3rd ‘snapshot’ were feeling less concerned about their diabetes due to COVID-19. They were also feeling less alone with their diabetes. Our findings likely reflect a reduction in the number of COVID-19 cases in the community at this timepoint, the easing of restrictions and people re-engaging socially.

Younger adults (18-34 years) appear especially impacted emotionally and feeling ‘burned out’ was most common at the 3rd ‘snapshot’. This is consistent with Australian findings among younger adults with type 2 diabetes (11).

The strengths of this study are that these novel ‘snapshot’ data have enabled examination of how people with diabetes are feeling during three timepoints during the COVID-19 pandemic. Participants were receptive to being asked by NDSS Helpline staff about their emotions related to diabetes, and referral options (i.e. factsheets and ‘on-call health professionals’) were in place for those experiencing distress. This is encouraging given that most callers were phoning the NDSS Helpline for practical support with the management of their diabetes.

Limitations include the cross-sectional study design, which precludes inferences about the impact of the pandemic on distress within participants over time. Around 20% of NDSS Helpline callers were invited to participate and most accepted. However, it is unclear whether their experience of diabetes concerns related to feeling ‘burned out’, ‘alone’, and ‘worried due to COVID-19’ can be generalised to those who were not invited to participate, nor to the general adult population living with diabetes. This is particularly pertinent given the higher number of adults with T1D who participated in our surveys and the under-representation of adults with T2D. Another limitation is the selection of only three PAID items, which limits insights into diabetes distress typically captured by a 20-item measure. It is possible that participants may have been experiencing general or diabetes distress or mental health problems not captured by these survey items.

In conclusion, the three ‘snapshots’ suggest that diabetes distress, specifically associated with feeling ‘burned out’, ‘alone’, and ‘worried due to COVID-19’, are significant issues for adults with diabetes in Australia during COVID-19. Prospective data are needed to improve understanding of the trajectory of the emotional impact of COVID-19 on people with diabetes and to inform when and how to target support for those who need it most.

## Data Availability Statement

Data was collected by the Australian National Diabetes Services Scheme (NDSS). De-identified raw data was provided to the co-authors. Requests to access the data would need to be approved by the NDSS. Requests to access the datasets should be directed to eholloway@acbrd.org.au.

## Ethics Statement

Ethical review and approval were not required for the study on human participants in accordance with the local legislation and institutional requirements. Written informed consent for participation was not required for this study in accordance with the national legislation and the institutional requirements.

## Author Contributions

JS and CH conceived of the project with input from TS, EH, and GC. EH and GC coordinated the surveys. EH conducted the data analysis. EH prepared the first and subsequent drafts of this manuscript, following co-author review. All authors reviewed and approved submission of the final manuscript.

## Funding

The ‘snapshot’ surveys were conducted in 2020-21 as part of the NDSS Mental Health and Diabetes National Priority Area, and funded by the NDSS. The NDSS is an initiative of the Australian Government administered by Diabetes Australia. CH and JS are supported by core funding to the Australian Centre for Behavioural Research in Diabetes provided by the collaboration between Diabetes Victoria and Deakin University.

## Conflict of Interest

GC is the manager of the NDSS Helpline, and JS is the Leader of the NDSS Mental Health and Diabetes National Priority Area.

The authors declare that the research was conducted in the absence of any commercial or financial relationships that could be construed as a potential conflict of interest.

The reviewer AS declared a past co-authorship with the authors to the handling editor.

## Publisher’s Note

All claims expressed in this article are solely those of the authors and do not necessarily represent those of their affiliated organizations, or those of the publisher, the editors and the reviewers. Any product that may be evaluated in this article, or claim that may be made by its manufacturer, is not guaranteed or endorsed by the publisher.
